# Bisphenol A (BPA) Modifies Cancer Signaling Pathways: A Neglected Global Health Threat

**DOI:** 10.3390/jox15060207

**Published:** 2025-12-04

**Authors:** Minatullah Al-Ani, Yassir Al-Ani, Shahad Sabaawi Ibrahim, Raghad Sabaawi Ibrahim, Peter Kubatka, Dietrich Büsselberg

**Affiliations:** 1 Weill Cornell Medicine-Qatar, Education City, Qatar Foundation, Doha 24144, Qatar; mia2041@qatar-med.cornell.edu (M.A.-A.); ssi4001@qatar-med.cornell.edu (S.S.I.); rsi4001@qatar-med.cornell.edu (R.S.I.); 2 Faculty of Medical Sciences, Newcastle University Medicine Malaysia, NUMed Malaysia Campus, EduCity, Iskandar Puteri 79200, Johor, Malaysia; y.a.a.al-ani2@newcastle.edu.my; 3 Laboratory of Experimental and Clinical Regenerative Medicine, Small Animal Clinic, University of Veterinary Medicine and Pharmacy in Kosice, 04181 Kosice, Slovakia; peter.kubatka@uvlf.sk

**Keywords:** Bisphenol A, endocrine disrupting chemicals, cancer signaling pathways, estrogen receptor pathway, GPER pathway, Bisphenol S, Bisphenol F, Bisphenol AF

## Abstract

Bisphenol A (BPA), a synthetic industrial compound widely found in plastics and other materials, has been linked to cancer development. As human exposure increases, BPA may pose potential carcinogenic concerns. Although BPA binds to estrogen receptors with much lower affinity than natural estrogens, its accumulation in human tissues can cause harmful effects. This review summarizes current evidence on BPA’s role in cancer initiation and progression, with a focus on its effects on cancer signaling pathways. These effects involve modulating pathways involved in cell growth, movement, invasion, survival, and adhesion. BPA acts as an estrogen ligand, binding to estrogen receptors and activating related pathways. The main route of exposure is through dietary intake of canned and plastic-packaged foods, with migration rates increasing at higher temperatures. To raise awareness of BPA’s harmful effects, industries have proposed “BPA-free” alternatives, some of which use derivatives like bisphenol S (BPS) and bisphenol F (BPF), which, unfortunately, may have even worse effects on human health. Given the ongoing challenges of eliminating BPA and similar harmful compounds, future research should focus on identifying safe substitutes, developing more effective removal technologies, and strengthening stringent regulations to mitigate public health risks.

## 1. Introduction

### 1.1. Plastics and BPA

Since the 1950s, plastics have been increasingly used as raw materials and in industrial production [1]. With today’s fast-paced lifestyles, improved standards of living, and economic growth, food preparation and consumption habits have driven unprecedented levels of plastic use, especially in packaging and single-use items [2,3]. BPA, one of the most ubiquitous industrial endocrine-disrupting chemicals (EDCs) that are inherently present in plastic materials [4], was identified as one of the five most frequently detected plastic-associated chemicals leaching from food contact materials [5], particularly after exposure to water, heat, or UV light. BPA is also used in the manufacturing of plastics and epoxy resins, ultimately for making plastic bottles, food packaging, toys, and other household items. Annually, approximately 15 billion pounds of BPA are produced [6], and this figure is projected to increase due to a globally anticipated compound annual growth rate (CAGR) for BPA.

### 1.2. BPA’s Routes of Exposure

Dietary exposure to BPA is the primary route by which the compound enters the human body. Plastic and canned food containers are among the primary sources of BPA exposure; however, consuming seafood or freshwater fish contaminated with BPA can also lead to the same effects [7]. BPA in polluted water can enter the bodies of plant and animal species, leading to ecological and health problems, especially after bioconcentration and biomagnification of the compound. BPA concentrations in water bodies worldwide have been monitored. In China, one of the world’s largest plastic producers, levels in river water and lake sediments were alarmingly high, ranging from 4 to 270 ng·g^−1^ [8]. Polluted water can increase the concentration of BPA in soil [9]. Similarly, the enrichment of industrial sludge, which contains BPA-contaminated atmospheric disposal, is also one of the main routes by which the compound enters the soil. Soil, which is one of the primary substrates of life, represents the main reservoir of BPA accumulation, as it supports plant species. It also plays a vital role in the biomagnification of the compound, as BPA can be released from the plastic matrix in which it is contained and then migrate through water or subsoil [10]. Therefore, BPA can be directly transferred to humans through exposure or leaching into the environment, such as seawater or soil.

### 1.3. BPA’s Mechanism of Action

**Figure 1:**
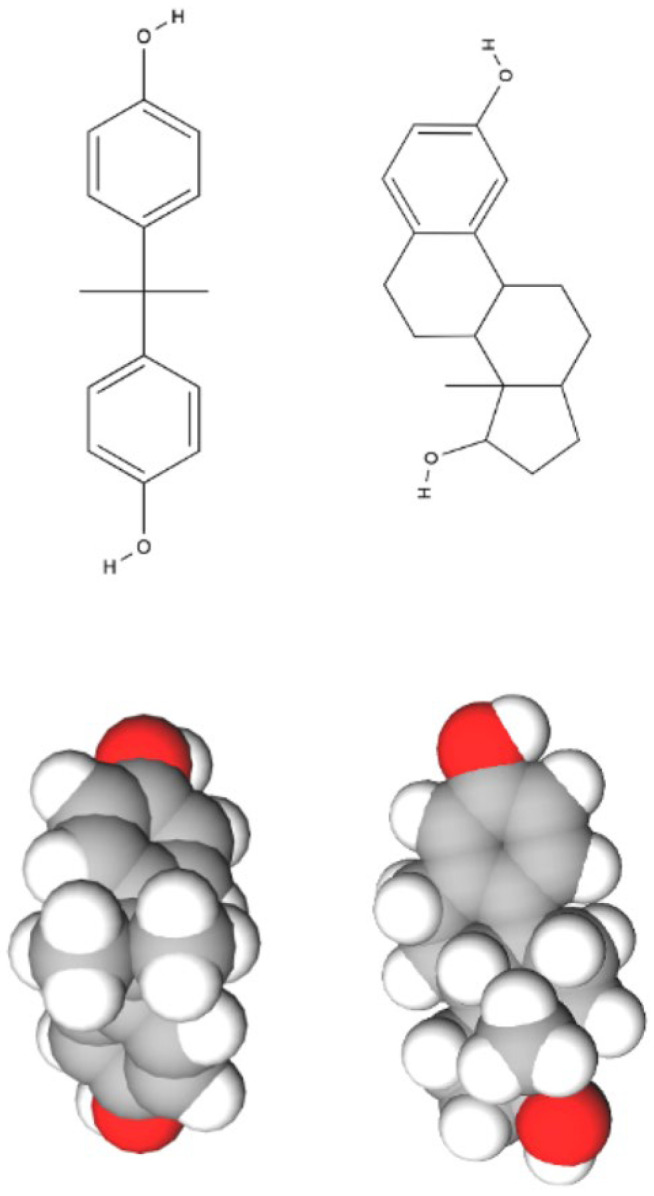
The ability of BPA to activate ERα/β is justified by the similarity in molecular structure between BPA (on the left) and 17β-estradiol (E2) (on the right). BPA (C_15_H_16_O_2_) and E2 (C_18_H_24_O_2_), a steroid hormone and an estrogen, are recognized by 2 hydroxyl groups at each end of the molecule. Atoms are colour-coded according to standard CPK convention: carbon (grey), hydrogen (white), and oxygen (red). Generated using MolView v2.4.

BPA’s mechanism of action includes mimicking estrogenic effects both in vivo and in vitro, despite lacking structural homology with the hormone estradiol [11]. BPA’s structural similarity to estrogen, however, allows it to compete with it for estrogen receptors α and β [12] (see Figure 1). BPA also binds to estrogen-related receptor gamma (ERRγ), a member of the orphan nuclear receptor family [11]. Studies have shown that with increasing doses of BPA, anti-estrogenic effects can take place. For instance, estrogen-dependent hippocampal synaptogenesis is reduced in a dose-dependent manner in some rat species. Significant inhibitory effects were observed at doses below the U.S. Environmental Protection Agency reference dose for human exposure. Hence, BPA may have antagonistic effects on estrogenic pathways, which may be exacerbated by increasing doses [13,14]. BPA interacts with additional nuclear receptors, including PPARγ and androgen receptors. Non-genomic signaling occurs through membrane-associated ERs, GPER-dependent rapid signaling, and cross-talk with EGFR and IGF-1R pathways.

**Figure 2:**
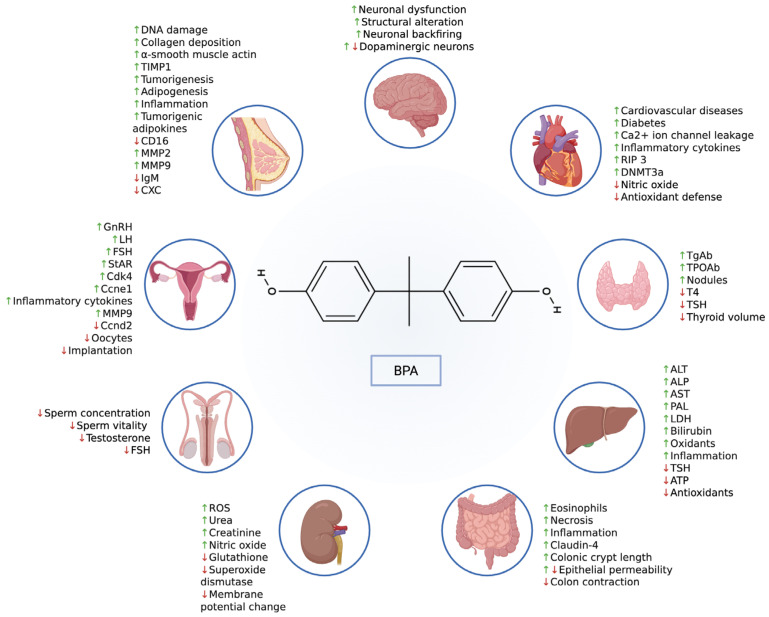
Effects of BPA on organs. BPA increases DNA damage, tumorigenesis, adipogenesis, inflammation, and collagen deposition in breast tissue. Similarly, it also contributes to inflammation in the intestines, liver, and uterine tissue in females. BPA decreases male fertility by reducing sperm concentration, sperm vitality, testosterone, and follicle-stimulating hormone levels (FSH) in males. For females, BPA increases levels of GnRH, LH, and FSH. In the kidney, BPA increases ROS, creatinine, and urea levels, indicating its ability to damage and alter organ function. Furthermore, BPA increases the risk of cardiac disease, diabetes, neuronal dysfunction, neuronal backfiring, structural brain alterations, bowel necrosis, bowel eosinophilia, elevated liver enzymes and bilirubin levels, thyroid nodules, and thyroid antibodies. Green upward arrows indicate increased expression or activation, whereas red downward arrows indicate reduced expression or functional decline. Generated using BioRender (https://www.biorender.com/) accessed on 26 November 2025.

BPA’s estrogenic effects have been recognized by the European Chemicals Agency (ECHA), which has deemed it a substance of very high concern (SVHC) after several experiments revealed that it mimics the natural hormone E2, leading to developmental, reproductive, and malignant diseases, especially when it interferes with endogenous periods during fetal life [15]. Other than its main endocrine-disrupting effect established through binding to ERs, BPA also binds to several different receptors involved in cancer signaling pathways, including androgen receptor (AR), G protein-coupled estrogen receptor (GPER; G protein-coupled receptor 30, GPR30), insulin-like growth factor-1 receptor (IGF-1R), and estrogen-coupled receptor gamma (ERRc) [16]. Although BPA interacts with several different receptors, it acts in a cell- and tissue-selective manner. Therefore, BPA may exhibit estrogenic activity in some tissues and not others, which may explain the varying levels of sensitivity between different organs to BPA exposure [17]. BPA can also act through non-estrogenic signaling pathways and has an affinity for other nuclear hormone receptors, which have adverse effects on many physiological functions, including spermatogenesis, TH homeostasis, stress response, and lipid metabolism (see Figure 2) [18]. BPAF exhibits higher ERα affinity than BPA (full agonist). BPS/BPF display similar potency but longer environmental persistence. BPM and BPP more strongly activate PI3K/Akt, enhancing EMT and metastasis.

Human exposure to BPA has also been associated with various cancers, including breast, ovarian, thyroid, prostate, colorectal, and lung cancer (see Table 1, Table 2 and Table 3). New BPA analogues, including Bisphenol F and Bisphenol S, have been commercially advertised as “BPA free”; however, several studies have linked these alternative compounds with tumor-promoting processes (see Table 4) [19,20]. Due to its ability to bind to estrogen receptors (ERs), BPA is mainly involved in the development of hormone-related cancers. The compound promotes the progression of estrogen-related tumors, especially in breast and ovarian cancers [21,22]. The occurrence and development of Ovarian Cancer are closely linked to exposure to EDCs; results suggest that various environmental chemicals, including BPA, are associated with a previous diagnosis of ovarian cancer among all women [23,24]. BPA affects receptors and gene expression, and it also induces transcriptional modifications linked to cancer-driving phenotypes in fibroblasts [25].

### 1.4. Prevalence of Human Exposure to BPA

Despite growing concerns about plastics and associated EDCs, demand for synthetic polymers derived from BPA continues to increase, and its prevalence in human tissues and urine is a testament to its widespread use (see Table 5). Although populations worldwide exhibit detectable blood and urine levels of BPA [26], certain groups may be exposed to significantly higher levels than others. Emerging adults (ages 18–25) in the United States had higher BPA exposure than adults aged 26 years and older. This difference was attributed to their unique lifestyles and behaviors, particularly their unhealthy diets and use of personal care products [27]. Variability is not limited to different age groups. Other countries have data to support differences in median urinary BPA levels and changes over time. In Canada, the median urinary BPA levels have remained almost the same over five years (1.1–1.2 ng/mL) while US levels decreased (1.9 to 1.3 ng/mL) and Korean levels increased (0.7 to 1.1 ng/mL) [28,29]. These differences can be used to assess trends in exposure to these chemicals across regions and to make necessary changes accordingly. A study conducted in the United States also found an inverse correlation between BPA exposure and family income. People who reported very low food security and received emergency food assistance had higher BPA concentrations than those who did not. There was also a positive correlation between being born in the U.S. and having a higher BPA concentration among Mexican Americans. National Health and Nutrition Examination Survey (NHANES) 2023–2024 analyses have not yet been published; however, NHANES 2003–2016 reports show an association between BPA and CVD, metabolic diseases, and hyperlipidemia. Detection in more than 90% of participants, with higher levels in low-income groups [27]. Korean and Chinese cohorts reported rising urinary BPS/BPF with concurrent declines in BPA, highlighting exposure-shifting.

### 1.5. Economic Burden on Healthcare

Plastics and their associated chemicals, particularly BPA, incur significant economic costs due to their adverse effects on human health. In 2015, the health-related costs of plastic production were estimated to exceed $250 billion globally. In the United States alone, the health costs associated with diseases caused by BPA and other chemicals linked to plastic exceeded $920 billion [30]. As BPA was found in the urine of nearly all people tested, it affects the health of the entire population. Even prenatal exposure can harm the health of infants and children [30]. BPA has been associated with reproductive abnormalities, cardiovascular risks, obesity, metabolic disorders, epigenetic alterations, autism, and various cancers [31]. BPA has detrimental effects at varying doses; at low doses, it influences development, metabolism, and behavior, while at high doses, it may lead to organ failure, leukemia, and severe weight loss [32].

## 2. Materials and Methods

This paper is a literature review of peer-reviewed scientific studies that examine the effects of BPA and its derivatives on cancer signaling pathways. A search strategy was employed using the PubMed database. The keywords used in the search included “BPA”, “Bisphenol A”, “Cancer”, and “Signaling Pathways”. Although the search was limited to studies from the past five years, many of the recent papers referenced older studies, which were subsequently reviewed and included to provide foundational support and context. The search (“BPA and cancer and pathway”) yielded 126 results from the past five years. After initial screening, 60 studies were selected and used to build summary tables. Only the most relevant were retained for full review.

## 3. Results

BPA Exposure and Its Carcinogenic Potential Across Cancer Types

Findings from in vitro and in vivo studies reveal that BPA enhances proliferation, migration, invasion, and epithelial–mesenchymal transition (EMT) in a wide range of cancer cell types, particularly hormone-sensitive cancers such as breast and ovarian cancer. These effects are mediated through classical and non-classical pathways, including estrogen receptors (ERα/ERβ), G protein-coupled estrogen receptor (GPER), EGFR, PI3K/AKT, MAPK/ERK, and STAT3. BPA analogues, such as BPF, BPAF, and BPS, despite being marketed as safer alternatives, exhibit tumor-promoting properties that are similar or even more potent. Comprehensive reviews indicate that BPS and BPF have equivalent estrogenic potency to BPA, affecting organ development and enzyme expression; BPAF often exceeds BPA in ERα activation and gene regulation.

**Figure 3:**
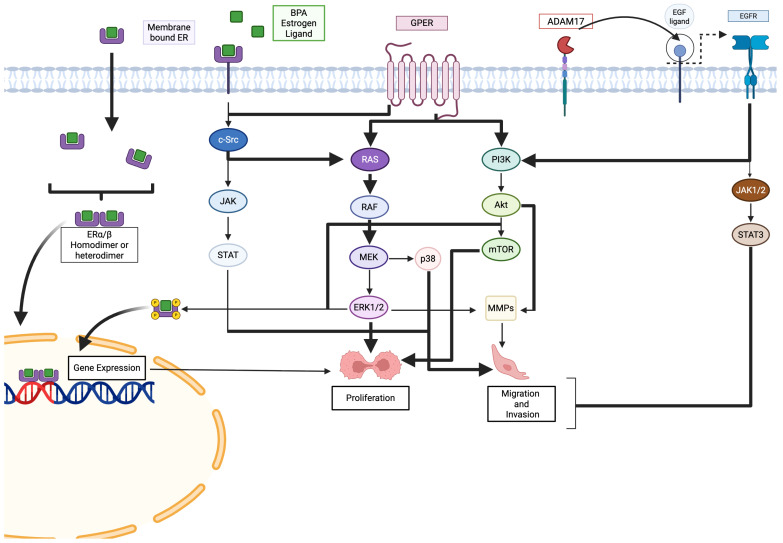
BPA increases cell proliferation, cell migration, cell invasion, and gene expression via ER and GPER receptor-related pathways. BPA activates gene expression by forming homodimers or heterodimers with ER receptors. BPA can also interact with EGFR and GPER receptors, activating PI3K/Akt signaling pathways and driving gene expression. Arrows represent the transmission of intracellular signaling, where one component exerts an activating influence on the subsequent effector in the cascade.

Overall, the data support a consistent pattern of BPA-induced dysregulation of cancer-related gene expression and signal transduction, including telomerase activation, altered cyclin expression (CCND1, CCNE1), increased N-cadherin/E-cadherin ratio, and ROS-mediated DNA damage induced by BPF/BPAF, often occurring even at low or environmentally relevant concentrations. These results underscore BPA’s under-recognized role as a carcinogenic risk factor and highlight the need for stricter regulatory oversight and continued investigation into its molecular effects (see Figure 3).

BPA activates the ER-related JAK/STAT pathway. BPA interacts with GPER-related pathways, such as the RAS/RAF/MEK pathway, to trigger proliferation via ERK1/2 signaling and migration and invasion via p38 signaling. Generated using BioRender.

### 3.1. Breast Cancer

BPA enhances cell viability in MCF-7 cells at concentrations as low as 10^−7^ mol/L [33] and SK-BR3 (HER2+) and MDA-MB-231 (triple-negative), when treated with concentrations between 10^−10^ and 10^−6^ M [34]. Even when treated under hypoxic conditions, BVECs, SKBR3, and MDA-MB-231 cells show increased viability [35]. BPA also induces ERα and proliferation markers, such as proliferating cell nuclear antigen (PCNA), at low doses (50 and 100 nM) in the same cell lines mentioned above [33]. In MCF-7 cells, BPA upregulates genes involved in the cell cycle and downregulates anti-proliferative genes, thereby influencing the G1/S transition through ERα signaling and promoting further cell proliferation [36]. Therefore, through its effects on cancer signaling pathways, including the GPER and EGFR/ERK pathways, BPA can increase apoptosis [37] and gene expression [35,38], thereby promoting cell viability and proliferation in breast cancer cell lines [35,38].

Repeated exposure to 50 and 100 nM BPA in MCF-7 cells enhances viability and significantly increases cell migration by more than twofold through an ERα-dependent pathway, while also increasing invasion potential [33]. At concentrations of 0.1–2.0 μM over 48 h, BPA further promotes migration and invasion in MCF-7 cells through GPER-mediated signaling [39]. Similarly, in 4T1 murine TNBC cells, exposure to 0.4, 1, and 2 μM BPA over 24 h also enhances migration and invasion, with GPER expression identified as a central mechanism [40]. GPER/GPR30 is a membrane GPCR that induces cell proliferation through activating ERK1/2 and protein kinase A [41]. SKBR3 cells and CAFs exhibit a similar pattern of GPER-dependent mechanisms [42]. Through EFGR transactivation, MDA-MB-231 cells also show increased migration [43]. BPA’s effects on migration are also seen under hypoxic conditions [35].

In addition to the GPER and EFGR pathways, BPA can also act by activating vascular endothelial growth factor (VEGF), leading to angiogenesis in breast tumors, activating STAT3 signaling, and activating the MAPK signaling pathway [44]. BPA may also depend on Esr1 and HDAC6 to promote breast cancer and may further lead to the activation of oncogene c-Myc in Osteosarcoma [45] (See Table 1).

**Table 1:** Effects of Bisphenol A (BPA) on breast cancer in in vitro studies.

Subtype	Cell Line	Dose of BPA and Exposure Time	Signaling Pathway and Target Gene	Results	Effect of Agonist/Antagonist	Reference
Estrogen receptor positive	MCF-7	0.01–1 μM of BPF and 0.001–1 μM of BPAF	↑ERα and GPER1-mediated signaling pathways ↑MAPK ↑PI3K/Akt	↑cell viability at lower doses ↓cell viability at higher doses ↑ROS ↑intracellular calcium ↑Cyclin D ↑c-myc	↑Effect with ERα agonist (PPT); no effect with ERβ agonist (DPN)	[46]
	MCF-7	10^−7^ to 10^−5^ M for 6 days	↑ERα	↑cell proliferation ↑progesterone receptor ↑cyclin D1 ↑G1/S transition ↓p21		[36]
	MCF-7	10^−9^ M (1 nM) for 24–48 h	↑Erα ↑HOXB9 ↑phosphorylated ERK1/2 ↓p21	↑cell proliferation	HOXB9 silencing via siRNA ↓BPA-induced proliferation	[47]
	MCF-7	10^−9^ M BPA for every 24 h (up to 200 days)	↑TNFα/NF-κB signaling	↑epithelial–mesenchymal transition ↑cell proliferation ↑cell viability ↓E-cadherin expression ↑ATP concentration ↑migration ↑invasion		[33]
Triple-Negative Breast Cancer (TNBC)	TNBC 4T1	0.4, 1 and 2 μM BPA for 24 h and 1 μM BPA for 12, 18 and 24 h	↑GPER	↑MMP-2 ↑MMP-9 ↑cell migration ↑cell invasion		[40]
	TNBC MDA-MB-231	1 μM BPA for up to 48 h of treatment	↑FAK ↑Src ↑ERK2 via GPER ↑EGFR transactivation pathway	↑cell migration ↑focal adhesions assembly via GPER/EFGR	GPER knockdown with siRNA EGFR inhibitor (AG1478): Inhibit BPA-induced effects	[43]
7	MDA-MB-231 MCF10A MCF12A	0.1, 0.8, 1, 2, and 3 μM for up to 48 h of treatment	↑GPER ↑p-FAK ↑Src ↑Ras/MEK/ERK1/2	↑cell migration ↑cell invasion ↑cell proliferation ↑AP-1-DNA binding ↑NFκB-DNA binding ↑p50 ↓IκBα	GPER siRNA FAK (FRNK), Src (PP2), and ERK2 inhibitors block BPA-induced effects	[39]
HER2-Positive ER-negative	SKBR3 CAF	1 μM for up to 48 h of treatment	↑ER ↑GPER/EGFR/ERK ↑c-FOS ↑CTGF	↑cell proliferation ↑cell migration ↑ERK1/2 phosphorylation	GPER silencing (shGPER) EGFR and MEK inhibitors blocked BPA induced effects	[42]
	MMTV-erbB2	0, 50, 500 ng/kg and 250 µg/kg	↑ER and erbB2 ↑ERα, cyclin D1, and c-myc ↑EGFR, erbB-3, Erk1/2, and Akt ↑ERα ↑Bcl-2 ↑RTK signaling	↑cell proliferation ↑ER-EGFR/erbB2 crosstalk		[48]
Multiple Subtypes (IBC), ER-negative	SUM149 SUM190	1 nM, 10 nM, 40 nM, 10 μM for up to 3 weeks	↑EGFR/HER2 ↑NF- κB	↑cell proliferation ↑tumor growth ↑SOD1 ↑Bcl-2 ↓apoptosis	EGFR inhibitor (GW583340/lapatinib analog)	[38]
	MCF-7 SK-BR3 MDA-MB-231	10^−8^ M for 30 days	↑VEGF/VEGFR ↑HIF signaling pathways	↑cell viability ↑cell proliferation ↑cell migration ↑IL19, CA9 and SPARC ↑NKT, NK and T cell		[34]
	BVEC SkBr-3 MDA-MB-231	150 mg/kg	↑HIF-1α/VEGF ↑GPER	↑cell migration ↑cell proliferation ↑cell viability ↑tumor growth		[35]

*Upward arrows indicate increased expression or activation, whereas downward arrows indicate reduced expression or functional decline.*

### 3.2. Ovarian Cancer

In Ovarian Cancer Cell lines SKOV3 and A2780, BPA concentrations of 10 and 100 nM increased migration and invasion as well as epithelial-to-mesenchymal transition (EMT) [49]. Similarly, in OVCAR-3 cells, BPA also stimulated migration by upregulating migration-associated factors, including MMP-2, MMP-9, and N-cadherin, at concentrations of 40 and 100 nM for 24 h [50]. One pathway that stimulates proliferation, migration, and invasion upon exposure to 10 μM BPA is the ERα/AKT/mTOR/HIF-1α signaling axis. Activation of this pathway enhanced glycolytic activity, including increased glucose uptake, lactic acid release, and intracellular ATP production [51]. Overall, OVCAR-3 cells treated with 1–100 nM BPA showed stimulated migration, invasion, adhesion to vascular endothelial cells, and proliferation, all of which were mediated by ERα signaling [52]. Other pathways, including STAT3, ERK1/2, and AKT, also promoted proliferation after BPA exposure in the same cell lines [53]. Migration and metastasis-associated markers, such as vimentin, cathepsin D, and MMP-2, were upregulated in BG-1 cells after BPA treatment, whereas E-cadherin was downregulated through ER signaling. This led to suppression of the TGF-β signaling pathway and increased cell migration [54]. As with OVCAR-3, SKOV3, and A2780 cell lines, BPA doses up to 100 nM also increased cell proliferation and estrogen response element (ERE) activity in BG-1 cells.This increase in proliferation and cell growth is due to the upregulation of ERα and IGF-1R mRNA levels, driven by the estrogenic effects of BPA [55]. Another way BPA stimulates proliferation and promotes growth in other ovarian cancer subtypes, such as granulosa cell tumor COV434 cells, is by modulating the expression of adipokines that act on ovarian cancer cells. BPA also disrupted cellular homeostasis by inducing apoptosis via ADAM17 activity and activating several downstream signaling pathways, including those mediated by G-protein-coupled receptors and tyrosine kinase receptors (See Table 2).

**Table 2:** Effects of Bisphenol A (BPA) on Ovarian Cancer in in vitro studies.

Subtype	Cell Line	Dose of BPA and Exposure Time	Signaling Pathway and Target Gene	Results	Reference
**Serous Carcinoma**	SKOV3 cells A2780 cells	10 or 100 nM for 24 h	↑Canonical Wnt Pathway ↑PI3K/Akt	↑migration ↑invasion ↑β-catenin translocation to nucleus ↑miR-21 ↑miR-222 ↑EMT ↑MMP9 ↓SERPINB5 (maspin) ↓TIMP3 ↓ZO-1	[49]
	OVCAR-3	0.1, 10, 40, and 100 nM for 3, 6, 24, and 48 h	↑MAPK ↑PI3K/Akt	↑cell migration ↑MMP-2 ↑MMP-9 ↑N-cadherin	[50]
	OVCAR-3	0.2, 2, 8 and 20 ng/mL for 24 h	↑Leptin ↑p-Stat3 ↑ERK1/2 ↑p-Akt.	↑cell proliferation	[53]
	SKOV3 A2780	10 nM/100 nM/1 μM/10 μM/100 μM/1 mM for 24 h	↑PINK1 ↓p53	↑OCT4 ↑NANOG ↑SOX2 ↓TOM20 and TIM23 ↑mitophagy	[19]
	OVCAR-3	10^−5^, 10^−6^, 10^−7^, 10^−8^, 10^−9^, 10^−10^ M for 24 h	↑WNT/β-catenin pathway	↑ALDH1A1 ↑CD133 ↑SOX2 ↑NANOG ↑OCT4 ↑CD44+CD24−	[56]
	ES-2 OVCAR-3	0.1, 1, 10, and 100 μM	↑ERα/AKT/mTOR/HIF-1α ↑PI3K-AKT	↑proliferation ↑migration ↑invasion ↑HIF-1α ↑GLUT3 ↑LDHA ↑lactate release ↑ATP production ↑Glycolysis	[51]
	OVCAR-3	1, 10 or 100 nM for 24 h or 5 days	↑ERα	↑Proliferation ↑Migration ↑Invasion ↑adhesion ↑MMP-2 andMMP-9	[52]
**Endometroid Carcinoma**	BG-1	10^−6^ M	↑ER pathway ↑c-Fos gene ↓TGF-β	↑cell proliferation ↑tumor volume ↑BrdU-positive nuclei ↑SnoN ↓p- Smad3	[57]
	BG-1	10^−6^ M for 1–48 h	↑ER pathway ↓TGF-β	↑cell migration ↑SnoN ↑vimentin ↓metastasis ↓E-cadherin ↓p- Smad3	[54]
	BG-1 A2780	10^−9^ to 10^−5^ M for 24 h	↑p38 MAPK ↑ERK 1/2	↑cell proliferation ↑tumor growth	[58]
	BG-1	10^−5^ M for 5 days	↑ERα ↑IGF-1R	↑cell proliferation ↑cell viability ↑p-IRS-1 ↑p-Akt 1/2/3 ↑cyclin D1	[55]
**Multiple**	OVCAR-3 SKOV-3 OV434 KGN	1–20 nM in human serum	↑ER signaling pathways	↑cell proliferation ↑leptin mRNA ↑PPARγ ↓adiponectin ↓chemerin ↑PPARγ	[59]
		100 fM to 100 mM for 24 to 72 h	↓PTEN ↑PI3K/Akt	↑p-AKT(Thr308) ↑p-AKT(Ser473) ↑apoptosis ↑follicular atresia ↓cell viability ↓primordial follicle pool (Ser473)	[60]

*Upward arrows indicate increased expression or activation, whereas downward arrows indicate reduced expression or functional decline.*

### 3.3. Other Cancers

In endometrial cancer cells such as HEC265, Ishikawa, and HEC151, BPA increased cell proliferation by promoting ERRγ translocation through both epidermal growth factor (EGF)-dependent and EGF-independent pathways. Different cell lines were affected through various pathways. For example, BPA induced cell proliferation via the BPA/ERRγ/EGF/EGFR/ERK signaling pathway in Ishikawa cells and through the BPA/ERRγ pathway in HEC265 cells. There was also a significant increase in cell viability; however, other cell lines, such as HEC251 (Grade II), HEC108 (Grade III), and HEC50B (Grade III), were not affected [61]. BPA also affects endocrine glands. In human thyroid cells, BPA exposure increased EMT and cell growth at concentrations from 10^−10^ M to 10^−4^ M in papillary thyroid cancer cells with the BRAFV600E mutation. BPA exposure at 10^−7^ M also increased migration, proliferation, and invasion of Nthy-ori 3-1 thyroid cells [62]. In the human adrenal cell line H295A, BPA increased cell proliferation by increasing levels of PCNA, cyclin D1, and cyclin D2. There was also an observed activation of the Shh pathway through an ERβ-mediated mechanism [63].

BPA also promoted proliferation and migration in colon cancer cells. In HT-29 cell lines, BPA exposure decreased PTEN expression, increased cellular stress, and triggered extrinsic apoptosis mechanisms, thereby contributing to elevated ERβ gene expression [64]. Exposure to BPA also activated the ERK pathway, increased expression of 5-HT3 receptors, and reduced E-cadherin expression, all of which could contribute to carcinogenesis and metastasis [65]. In other human colon cell lines, including epithelial (HCoEpiC) and colon cancer (HCT116) cells, BPA increased invasion at low doses (0.0043 nM) and exerted cytotoxic effects, reducing cell viability at high doses (1 and 10 µg/mL) [66].

Nasopharyngeal carcinomas (NPC) and cancers of the respiratory tract exhibit increased malignancy after exposure to BPA through β-catenin-mediated pathways. BPA enhanced the proliferation and migration of NPC cells [67] and induced DNA damage, cytotoxicity, and ROS production in human bronchial epithelial cells through ATM/ATR/p53/γ-H2AX-dependent phosphorylation [68].

BPA also impacts liver cancer, as seen in human hepatocellular carcinoma (Hep3B), where BPA induces degradation of hypoxia-inducible factor 1-alpha (HIF-1α) through the lysosomal pathway, thereby disrupting the cellular response to hypoxia [69].

Hematologic malignancies are also affected by exposure to BPA. In human lymphoblastoid cells (SUP-B15 and TK6), BPA disrupted the cell cycle and induced DNA damage by activating the CTNNB1 pathway. The compound contributed to lymphomagenesis by promoting the growth of clonogenic cervical cells in damaged cells and dysregulating DNA repair-associated genes TP53 and CDKN1A [70].

BPA also affects rare types of cancer. In human neuroblastoma cell lines (IMR-32 and SK-N-SH), exposure to BPA resulted in increased ROS production, LDH leakage, and apoptosis, leading to reduced cell viability and mitochondrial membrane potential in both cell lines [71] (See Table 3).

**Table 3:** Effect of Bisphenol A (BPA) on cancers other than breast and ovarian cancer in in vivo and in vitro studies.

Cancer Type	Cell Line	Dose of BPA and Exposure Time	Signaling Pathway and Target Gene	Results	Reference
Uterine leiomyoma	UL	10 μmol/L	↑ERα ↑IGF-1 ↑VEGF	↑cell proliferation ↑SnoN ↓p- Smad3 ↓c-fos protein	[72]
Uterine leiomyoma	Human UL cells	100 μL of medium with 103 μmol/L of E2 10 μmol/L of BPA 32 μmol/L of NP 8 μmol/L of octylphenol for 24–72 h	↑ERα signaling ↑IGF-1 ↑VEGF ↑Akt	↑cell proliferation ↑SnoN protein ↓p-Smad3 protein ↓c-fos protein	[73]
Hepatocarcinoma	Hep3B MCF-7 LM8	50–200 µM of BPA or BPAF under normoxia or hypoxia for 6 h	↑Lysosomal and Proteasomal Pathway	↑cell proliferation ↑HSC70 ↓HIF-1alpha ↓EPO mRNA	[69]
Adrenal cortical	H295A NCI-H295	1–1000 nM for 72 h	↑Shh pathway ↑ERβ-mediated activation	↑adrenal gland weight ↑cell proliferation ↑nuclear translocation of Erβ ↑cyclin D1 ↑cyclin D2	[63]
Lung	Lung tissue of adult female Wistar rats	150 mg/kg body weight/day for 6 weeks	↑EGFR ↑KRAS ↑ERK1/2	↑MMP-2 ↑MMP-9 ↑TNF-α, IL-6, IL-1β ↑GRP	[74]
Colon	HT-29	4.4 µM	↑Erα ↑miR-200c and miR-141	↓PTEN ↓ATR	[64]
Colon	HCoEpiC HCT116	0.0043 nM for 2 months	↑Wnt/β-catenin pathway	↑Invasion ↑Proliferation ↑Migration	[66]
Colon	HT-29	4.4, 6.6, 8.8 µM	↓FADD, FAS ↓APAF-1 ↓CASP2 ↓CASP9 ↓FOXO3 ↓P53	↑ESR1 ↑proliferation	[65]
Thyroid	BCPAP Nthy-ori3-1 Thyroid tissue of 3–4 weeks-old female SD rats	10–10 −5 × 10^−5^ mol/L and 50 μmol/L for 24 h and 48 h	↑ERK	↑β-catenin ↑HDAC6 ↓PTEN ↑c-MYC ↑proliferation ↑AKT signaling ↑Migration ↑phospho-Akt	[75]
Endometrial	HEC265	1 nM to 1 μM for 0.5, 1, 3, and 6 h and 24 h	↑activation of the EGFR/ERK pathway	↑cell proliferation ↑nuclear translocation of ERRγ ↑influx of Ca^2+^ ↑EGF secretion	[62]
Osteosarcoma	SaOs-2	0.1, 1, 10 μM	↑IL6/JAK2/STAT3	↑proliferation ↑migration ↑invasion ↑DLGAP5	[76]
Neuroblastoma	IMR-32 SK-N-SH cells	0, 1, 10, and 100 nM, and 1, 10, and 100 μM for 24 h	↑NLRP3/caspase-1/GSDMD	↑cell apoptosis ↑inflammation ↑IL-18, ASC, GSDMD ↑NLRP3, caspase-1 and GSDMD ↑ROS ↑LDH ↓mitochondrial membrane potential	[71]
Myeloblastic	Peripheral blood cells, kidney, liver and spleen tissue of embryos, larvae and adult zebrafish	100, 500 and 2500 μg/L for 96 hpf (larvae) 5 to 30 mg/L concentrations for 96 h. (adult)	↑EGFR/ERK signaling	↑cell apoptosis ↑binding affinity to zebrafish EGFR ↑EGFR	[77]
Lymphoma	TK6 SUP-B15	10, 103, 105 nM of BPA for 24, 48, and 72 h	↑CTNNB1	↑Survival of TK6 lymphoblastoid cells ↓TP53 ↓CDKN1A ↑NFKB1 ↑AR ↑IGF1 ↑TWIST1	[70]
Nasopharyngeal	NPC CNE2 CNE1 5–8F	10 nM BPA for 12 h, 24 h, 48 h	↑Wnt/β-catenin pathway	↑mRNA stability of β-catenin via miR-214-3 ↓pSer45 of β-catenin via CK1α ↑proliferation ↑migration ↑mRNA stability of β-catenin ↓miR-214-3p ↓phosphorylation of β-catenin ↓CK1α ↓CTNNB1	[61]
Bronchial and epithelial	BEAS-2B	12.5–200 μM for 24 h	↑ER/GPR30-ERK signaling pathway	↑ROS ↑DNA strand break ↑DNA tail formation ↑DNA histone damage ↑p-ATM/ATR complex ↑p-γH2AX	[68]
Reproductive Tract	SKOV3 BG-1 A2780 LNCaP	100 μM BPA 10, 50, or 100 μM NP for 24 h	↑ADAM17 ↑ERK pathway	↑apoptosis at higher doses ↑migration ↑intracellular calcium ↑cell proliferation	[78]

*Upward arrows indicate increased expression or activation, whereas downward arrows indicate reduced expression or functional decline.*

### 3.4. BPA Derivatives

BPAF is a halogenated fluorine-containing BPA derivative [79,80,81], which has significantly higher binding affinities to both ERα and ERβ than BPA and acts as a full agonist for ERα [82,83]. BPAF has been detected in human urine, serum, breast milk, placenta, and cord blood [84]. It exhibits estrogenic properties and therefore activates the ER pathway, inducing estrogen-like effects [85]. Low concentrations of BPAF have been linked to transcriptional changes and the disruption of reproductive organ development, leading to adverse outcomes in adulthood, particularly in males [84,86].

BPF, another BPA derivative, has been quantified in human plasma at levels 3 times higher than those of BPA [87]. This derivative has been linked to tumorigenesis progression and the initiation of invasive and metastatic phenotypes that are equivalent to, or even more deleterious than BPA [88,89]. BPF exposure in MCF-7 cells led to an increase in cell proliferation and viability, ROS, and Ca^2+^ at concentrations of 0.01–1 μM. BPF’s estrogenic activity activates PI3K/PKB and ERK1/2 signaling pathways through GPER1 in a manner comparable to BPA. This results in the upregulation of Erα, c-Myc, and cyclin D protein expression [90].

Exposure to BPS in MCF-7 breast cancer cells similarly contributes to mutation and gene expression changes associated with tumor development. BPS altered DNA methylation patterns in the promoter regions of cancer-related genes and in another cell line (TNBC), triggering cell migration via activation of the Hippo-YAP pathway through GPER/PKC-mediated inhibition of LATS1/2 [91]. Two other derivatives, BPP and BPM, both stimulate migration, invasion, EMT, and metastasis in TNBC cells, but do so through activation of the PI3K/AKT pathway [92]. BPS has also been identified as an EFGR agonist blocking cell proliferation in MDA-MB-231 cells and human cytotrophoblasts (hCTBs), leading to a decrease in EGF-mediated MYC expression [93]. In non-small cell lung cancer (NSCLC) cells, BPS exposure also promoted migration and invasion by upregulating TGF-β and activating the TGF-β/Smad2/3 signaling pathway [94]. See Table 4 and Table 5.

**Table 4:** Effect of Bisphenol A derivatives such as BPF, BPM, BPP, BPS on cancers, including breast, lung, and bladder in in vitro studies.

Cancer Type	Cell Line	BPA derivative and Concentration	Pathway	Results	Reference
Breast	Human breast cancer cells 4T1, 4T1-Luc and MDA-MB-231	5 mg/kg body mass via intraperitoneal administration of BPM, BPP, and BPA.	AKT	↑Invasion, ↑Migration, ↑EMT expression, ↑AKT phosphorylation	[89]
Breast	MCF-7 cells of the breast cancer cell line	Final concentrations of 1 μM, 100 nM, and 10 nM of BPS were used. Following a 48-h incubation to allow cell attachment, the medium was changed to include various concentrations of BPS.	N/A ^a^	↑Methylation in transposons of MCF-7 cells and most breast cancer-related genes, ↑Gene expression of breast cancer cells	[92]
Breast	MDA-MB-231 human breast cancer cells	BPS-10 (10 μg/kg body weight/day) and BPS-100 (100 μg/kg body weight/day)	N/A ^a^	↑Proliferation, ↑Deterioration, ↑Intratumor heterogeneity of lipid and protein distribution	[93]
Breast	MCF-7 cells of breast cancer cell line	Ranged from 0.00001 to 100 μM	ERα and GPER1	↑Cell proliferation, ↑Cell viability, ↑Intracellular ROS and Ca^2+^, ↑Protein expressions of ERα, GPER1, c-myc, and cyclin D, ↑Phosphorylation of PKB and ERK1/2	[46]
Breast	Human TNBC MDA-MB-231 and BT-549 breast cancer cell cultures	Nanomole (10^−9^ M) to millimole (10^−3^ M)	GPER/Hippo-YAP	↑Migration, ↓YAP and TAZ phosphorylation levels, ↑CTGF and ANKRD1, ↓Phosphorylation levels of LATS1/2	[88]
Includes breast	Human primary term cytotrophoblast cells (hCTBs) and MDA-MD-231 cells	0.0001 to 10 μg/mL	N/A ^a^	↓EGF binding, ↓EGF-mediated phosphorylated EGFR, ↓EGF internalization, ↓EGF-mediated hCTB syncytialization at 200 ng/mL of BPS	[90]
Lung	Human NSCLC cell A549, H1299 and H358 cell cultures	1, 10 and 100 nM of BPS,	TGF-β	↑Migration of NSCLC cells, ↑Vimentin, and MMP-2, ↑TGF-β expression and transcription, ↑Smad2/3 activation, ↑IL-8 expression in A549 cell, ↑IL-10 expression in H1299 cell	[91]
Bladder	RT4 Non-Invasive Bladder Cancer Cells, T24 Invasive Bladder Cancer Cells and normal Urothelial Cells	10^−8^ M BPA-gluc and 10^−8^ M BPS-gluc	N/A ^a^	↓Basal Glycolytic Capacity and migration of UCs, ↑Basal and maximal glycolytic capacity, mitochondrial respiration, and proliferation of RT4 cells, ↑Maximal glycolytic capacity and migration of T24 cells, ↑Proliferation of UCs	[94]

^a^ N/A: Data not available or not reported by the study.

**Table 5:** Experiments using Cancer Cells.

Type of Cancer	Pathway	Cell Line	Results	BPA/Derivatives Concentrations	Reference
Breast	AKT	Human breast cancer cells 4T1, 4T1-Luc and MDA-MB-231	↑Invasion, ↑Migration, ↑EMT expression, ↑AKT phosphorylation	5 mg/kg body mass via intraperitoneal administration of BPM, BPP, and BPA.	[89]
Breast	N/A ^a^	MCF-7 cells of the breast cancer cell line	↑Methylation in transposons of MCF-7 cells and most breast cancer-related genes, ↑Gene expression of breast cancer cells	1 μM, 100 nM, and 10 nM final concentrations of BPS were used. Following a 48-h incubation to allow cell attachment, the medium was changed to include various concentrations of BPS.	[92]
Breast	N/A ^a^	MDA-MB-231 human breast cancer cells	↑Proliferation, ↑Deterioration, ↑Intratumor heterogeneity of lipid and protein distribution	BPS-10 (10 μg/kg body weight/day) and BPS-100 (100 μg/kg body weight/day)	[95]
Breast	ERα and GPER1	MCF-7 cells of the breast cancer cell line	↑Cell proliferation, ↑Cell viability, ↑Intracellular ROS and Ca^2+^, ↑Protein expressions of ERα, GPER1, c-myc, and cyclin D, ↑Phosphorylation of PKB and ERK1/2	Ranged from 0.00001 to 100 μM	[46]
Breast	GPER/Hippo-YAP	Human TNBC MDA-MB-231 and BT-549 breast cancer cell cultures	↑Migration, ↓YAP and TAZ phosphorylation levels, ↑CTGF and ANKRD1, ↓Phosphorylation levels of LATS1/2	Nanomole (10^−9^ M) to millimole (10^−3^ M)	[88]
Includes breast	N/A ^a^	Human primary term cytotrophoblast cells (hCTBs) and MDA-MD-231 cells	↓EGF binding, ↓EGF-mediated phosphorylated EGFR, ↓EGF internalization, ↓EGF-mediated hCTB syncytialization at 200 ng/mL of BPS	0.0001 to 10 μg/mL	[90]
Lung	TGF-β	Human NSCLC cell A549, H1299 and H358 cell cultures	↑Migration of NSCLC cells, ↑Vimentin, and MMP-2, ↑TGF-β expression and transcription, ↑Smad2/3 activation, ↑IL-8 expression in A549 cell, ↑IL-10 expression in H1299 cell	1, 10, and 100 nM of BPS,	[91]
Bladder	N/A ^a^	RT4 Non-Invasive Bladder Cancer Cells, T24 Invasive Bladder Cancer Cells and normal Urothelial Cells	↓Basal Glycolytic Capacity and migration of UCs, ↑Basal and maximal glycolytic capacity, mitochondrial respiration and proliferation of RT4 cells, ↑Maximal glycolytic capacity and migration of T24 cells, ↑Proliferation of UCs	10^−8^ M BPA-gluc and 10^−8^ M BPS-gluc	[94]

^a^ N/A: Data not available or not reported by the study. *Upward arrows indicate increased expression or activation, whereas downward arrows indicate reduced expression or functional decline*.

## 4. Discussion

### 4.1. BPA and Cancer Signaling Pathways

BPA enhances proliferation, migration, and invasion in various cell lines by activating signaling pathways in cancer cells. Despite its structural similarity to estrogen, BPA exhibits a weak affinity for classical Estrogen Receptors (ERα and ERβ), 1000–2000 times lower than E2. Therefore, numerous studies have investigated the binding of BPA to other receptors, including GPER/GPR30, PPARγ, and ERRγ [15]. BPA’s function as an endocrine-disrupting chemical (EDC) explains its effects on hormone-related cancers, specifically Breast and Ovarian Cancers, especially after long-term low-dose exposure [21]. Emerging evidence indicates that BPA also disrupts signaling pathways in non-hormone-sensitive cancers, including colorectal, lung, and liver malignancies, by activating the EGFR/MAPK, PI3K/AKT/mTOR, and STAT3 pathways [66,69]. In colorectal cancer cells, for example, BPA induces the overexpression of GOLPH3, which promotes malignant behavior by activating the PI3K/AKT/mTOR cascade and ROS-driven HIF-1α/VEGF signaling, even under hypoxic conditions.

BPA’s effects on cell viability are dose-dependent; at low doses, it increases cell viability, whereas at higher doses, the opposite is true [61,66]. This is due to its estrogenic activity at lower concentrations and its cytotoxic effects at higher concentrations [68]. However, results show that BPA consistently promotes migration and invasion, even under hypoxic conditions [35]. Although its carcinogenic effects are similar, BPA acts through a variety of signaling pathways, including the estrogen receptor (ER) pathway, which is the most common in hormone-sensitive cancers [93]. This is because ERs are often overexpressed in the tissue of hormonal cancers, especially breast cancer, making cells susceptible to the action of BPA. One study found that BPA-exposed mice with mesenchymal ERα had a worse prognosis and overall survival than those without it, indicating that BPA acts on ERα and affects tumorigenesis [96]. Crucially, BPA is metabolized into 4-methyl-2,4-bis(4-hydroxyphenyl)pent-1-ene (MBP), an active metabolite with up to 1000-fold greater estrogenic activity and higher affinity for ERα/ERβ at nanomolar concentrations. MBP triggers ERK/Akt disruption, AMPK-mediated ER stress, and caspase-dependent apoptosis in lung and neuronal cells, suggesting potent pro-carcinogenic signaling in diverse tissues.

BPA derivatives, which have a similar chemical structure to BPA, are marketed as BPA-free but exhibit effects that are either similar to or more deleterious than BPA’s due to their estrogenic properties and are suspected to function as endocrine-disrupting chemicals (EDCs). BPF, BPAF, BPS, BPP, and BPM are compounds that exhibit estrogenic actions of a similar order of magnitude to BPA and have been linked to tumorigenesis in hormone-sensitive cancers, among other conditions [80]. Notably, in ovarian cancer cells, BPA activates the PI3K/Akt signaling pathway; analogues such as BPS and BPF display comparable estrogenic potency, while BPAF often exceeds BPA in ERα activation, suggesting their potential oncogenicity. Compared to its derivatives, kinetic analyses reveal that BPA is more susceptible to tissue accumulation due to its high potency, likely a result of its high lipophilicity and dissociation constant. BPS and BPF exhibit lower potency than BPA [97]. This may help explain differences in the metabolic effects of BPA and its derivatives. Within the AOP framework, BPA initiates molecular events (ERα/GPER binding, oxidative stress, PTEN suppression). Key events include aberrant PI3K/Akt activation, MAPK amplification, EMT promotion, and metabolic reprogramming, leading to adverse outcomes including tumor initiation and progression.

### 4.2. Health Risks Associated with BPA

BPA poses a significant risk to human health for several reasons, one of the main ones being its estrogenic activity. Even though its affinity for ERs is 10,000 to 100,000 times weaker than that of its natural counterpart, E2, its lipophilic nature enables it to accumulate in adipose tissue and cross the blood–brain barrier, maintaining a persistent presence in tissues long after exposure, and disrupt physiological functions, leading to detrimental health effects [98,99]. Interestingly, BPA exhibits stronger estrogenic activity at lower doses, as indicated by its higher activity at nanomolar concentrations compared to micromolar concentrations [100]. There are numerous health risks associated with BPA, as it negatively impacts reproductive health, the immune system, and metabolism. It is also linked to oncological diseases, as shown above, including hormone-sensitive cancers like breast, ovarian, and prostate cancer [101]. BPA is also associated with metabolic syndrome, obesity, insulin resistance, hypertension, and neurodevelopmental disorders [102].

Exposure to BPA is influenced by a range of factors, including air pollution, workplace conditions, and consumption habits. Still, packaging methods and storage conditions may be the most significant. Elevated temperatures increase the rate at which BPA migrates from plastics to food. High temperatures, such as those reached during boiling, can increase BPA leaching rates by up to 55-fold compared to room temperature, significantly raising the risk of exposure during cooking or microwaving [103]. In addition to plastic, food stored in canned materials absorbs higher levels of BPA than other packaged foods [104].

As of 2025, the EU maintains the strictest BPA restrictions. The EU bans BPA in food-contact products such as infant bottles (https://trade.ec.europa.eu/access-to-markets/en/news/eu-prohibition-use-and-trade-bisphenol-20-january-2025#:~:text=07%20March%202025-,EU%20prohibition%20on%20the%20use%20and%20trade%20of%20Bisphenol%20A,food%20contact%20materials%20and%20articles (accessed on 19 November 2025)). GCC nations follow EU standards in infant products. The US FDA maintains limited restrictions on BPA, which is considered safe at certain levels, and regulations on BPA differ by state (https://www.fda.gov/food/food-packaging-other-substances-come-contact-food-information-consumers/bisphenol-bpa-use-food-contact-application (accessed on 17 July 2025)).

Several precautionary measures can be taken to reduce or mitigate these risks, the most obvious being a limit on the use of plastic products. Some countries, including Japan, Canada, and members of the European Union, have already regulated the use of BPA by issuing specific bans on manufacturers and targeting particular consumer products to make them BPA-free [105]. These regulations have led to the rise of alternatives, including Bisphenol derivatives BPS and BPF. Although they may be “BPA-free”, the substitutes have not yielded encouraging results. Recent studies suggest these derivatives may be equally or even more harmful, promoting certain cancers and affecting reproductive health as well as other physiological functions [106].

Remediation and the removal of BPA from the environment remain other options. Specific conventional methods, such as coagulation, sedimentation, and filtration, have been deemed insufficient for effectively removing low-dose BPA contamination [107]. Economically attractive oxidation is another method that has considerable drawbacks, as it produces toxic byproducts and exhibits inadequate oxidation activity [108,109]. A promising biodegradable approach involves specific microbes, such as strains of Pseudomonas, Klebsiella, and Enterobacter, that can degrade up to 97% of BPA in a few days under optimal conditions. However, they often require supplementary treatment to ensure complete removal [110]. Ultimately, the total elimination of BPA from the environment is impractical and unrealistic, at least in the short term. Therefore, the best way forward is to reduce BPA exposure by using better alternatives and implementing improved waste management practices.

### 4.3. Clinical Relevance of BPA Exposure

#### 4.3.1. BPA’s Role in Chemoresistance

BPA protects cancer cells from chemotherapeutic-induced toxicity and causes chemoresistance, specifically to agents Doxorubicin, Cisplatin, and Vinblastine. These effects were observed in cell lines lacking ERα, confirming that BPA can act through non-classical receptors such as GPER/GPR30 or ERRγ. In estrogen-responsive T47D breast cancer cells, BPA also counteracted the cytotoxicity of the chemotherapeutic agents mentioned above and increased overall cell viability [111].

In other breast cancer chemotherapeutic agents, including selective estrogen receptor modulators such as Tamoxifen, BPA was also found to reduce cytotoxicity at low concentrations, specifically in MCF-7 cells. This was evidenced by the suppression of cell apoptosis, the transition of the cell cycle from G1 to S phase, and the upregulation of cyclin D1 and Erα. Within the same study, Tamoxifen was shown to induce apoptosis and inhibit cell cycle progression in MCF-7 cells, further confirming that the effect of BPA was indeed counteractive to tamoxifen’s cytotoxic effects in that cell line. Notably, BPA alone did not exhibit any inhibitory effects on apoptosis; relatively, it inhibited the apoptotic effect of tamoxifen in MCF-7 cells.

Mechanistically, BPA upregulates anti-apoptotic proteins BCL-2 and BCL-xL and activates the EGFR/ERK1/2 pathway, providing a survival advantage during chemotherapy, and this effect occurs at low nanomolar concentrations. This suggests that BPA not only triggers tumorigenesis but also may worsen prognosis by interfering with standard cancer treatment and promoting an anti-apoptotic phenotype. Researchers have proposed that BPA’s role in chemoresistance is anti-apoptotic rather than proliferative, as evidenced by chemoresistance even in the absence of estrogen [112]. Exposure to endocrine-active compounds like BPA could also subvert the effects of endocrine therapies like Tamoxifen, which reduce the risk of cancer recurrence, thereby promoting the growth and spread of estrogen receptor-positive tumors [113,114].

#### 4.3.2. Possible Treatments for BPA-Induced Carcinogenesis

Antagonists of estrogen receptors have been reported to reduce cell proliferation induced by BPA, G-15, and Fulvestrant (ICI 182 780, ICI), two drugs that have shown promising effects. Although G-15 is only a partial antagonist of GPER/GPR30, it successfully reduced BPA cell proliferation in vitro. Fulvestrant, on the other hand, when combined with low-dose BPA, not only synergistically decreased proliferation but also promoted cell migration and mesenchymal transition and switching from E-cadherin to N-cadherin expression in Hepg2 cells [115,116]. Fulvestrant but not G-15 also reversed the level of tissue Inhibitors of Metalloproteinases (TIMP) induced by BPA, which supports the suggestion that ERs mediate TIMP production rather than GPER [117]. Fulvestrant also attenuated the BPA-induced Th2 deviation, confirming the role of estrogen receptors (ERs) and BPA in anti-tumor immunity [118].

Other than the common antiestrogen drugs, natural substances have also had promising effects in reversing BPA’s carcinogenic effects. Naringenin, a flavonoid found in citrus fruits, antagonizes BPA-induced colon cancer cell proliferation and activates intrinsic apoptosis pathways via caspase-9, demonstrating its efficacy even in the presence of BPA [64]. Furthermore, naringenin inhibits the PI3K/Akt/mTOR and IL-6/STAT3 signaling pathways, key oncogenic routes frequently exploited by BPA to drive tumor progression. When exposed to both types of disruptors, naringenin and BPA, naringenin exhibits antagonistic properties on ERα, preventing the BPA-induced proliferation, similar to its synthetic counterparts, G-15 and Fulvestrant [119].

BPA derivatives like BPS, which have similar effects on carcinogenesis, can also be targeted through inhibitors. One pathway upregulated in TNBC cell lines after exposure to BPS is the Sonic Hedgehog (SHH) signaling pathway. Researchers found that a specific inhibitor of this pathway, Gli1 small interfering RNA (siRNA), reduced the effects of BPS on stemness, invasion, and migration of the cells [120]. Similarly, selective ERβ inhibition via siRNA reverses BPA-induced myofibroblastic transformation (α-SMA and collagen expression), highlighting ER subtype-specific blockade as a therapeutic strategy [121].

## 5. Future Perspectives

Preventive measures are crucial for mitigating the adverse effects of BPA on human health. The primary concern with BPA, along with other endocrine-disrupting chemicals (EDCs), is its bioaccumulation and incomplete degradation in the environment, which can contaminate ecological systems. One approach to addressing this issue is to convert EDCs into non-toxic, stable products via mineralization pathways [31,122]. Recent progress in integrated photocatalytic-biotic mineralization, employing iron-doped TiO_2_ or synergistic microalgae-bacteria consortia, has enabled near-complete degradation of BPA under simulated sunlight, highlighting its promising scalability for wastewater treatment facilities. Detecting BPA levels in soil and the atmosphere is essential to ensure that they do not exceed harmful concentrations and the daily tolerable intake. New portable technologies, such as molecularly imprinted electrochemical sensors (MIES) embedded in smartphone-compatible laser-scribed graphene platforms, now enable real-time, on-site detection of BPA with nanomolar-level sensitivity (limit of detection ~4 nM). In addition to preventive measures, regular health assessments should also monitor BPA levels to identify at-risk populations, provide necessary treatments, and implement targeted public health interventions to prevent transgenerational harm [123,124].

Another matter that requires further investigation is the impact on developing fetuses and infants, who may be particularly vulnerable to the effects of BPA. This compound is widespread in both developed and developing countries. This suggests that public education programs have not effectively informed at-risk populations about how to reduce their daily intake and absorption of BPA. The vulnerability of different populations to BPA may also result from co-exposure to other pollutants, which can lead to synergistic effects that warrant further studies to potentially mitigate the harmful impacts of BPA by exploring co-exposure patterns [125]. Priority research must investigate the combined toxic effects of BPA with phthalates, airborne particulates, and pesticide residues, employing high-throughput human stem cell-based assays to detect additive or synergistic risks from low-dose chemical mixtures.

Additionally, BPA alternatives should be monitored in the environment and studied to prevent the rise of “BPA-free” products, which contribute similarly to human disease. BPA alternatives have been previously researched; however, they have not been prioritized or studied systematically beyond reproductive studies. Despite the evidence that these alternatives can have long-term negative consequences that may be even more detrimental than BPA, they are not being recognized as serious threats to human health to the extent that they should be [126]. A recent multi-level high-throughput screening study assessed more than 30 bisphenol analogues and identified several, including BPZ and TDP, that exhibit significant estrogenic or anti-androgenic receptor activity, raising concerns about their safety profiles. Therefore, the regulatory identification and daily tolerable intake of BPA and its derivatives should be reviewed, and stricter industrial requirements must be implemented to prevent the harmful biological health effects of these compounds [123]. Regulations should enforce mandatory pre-approval testing of all new bisphenol analogues for endocrine disruption and genetic toxicity, utilizing validated in vitro and computational methods to ensure safety before market entry. There is also a need for more literature on practical, feasible ways to remove BPA from the environment, as well as a greater focus on developing safe BPA analogues that can truly be considered “BPA-Free” [119].

## 6. Conclusions

BPA is a pervasive EDC that intersects with key cancer signaling pathways across multiple organs. In vitro, in vivo, and epidemiological studies have consistently shown that BPA promotes proliferation, migration, invasion, EMT, metabolic reprogramming, and chemoresistance through ERα/ERβ, GPER, EGFR/MAPK, PI3K/AKT/mTOR, JAK/STAT, and many other cascades. The effects of BPA are exacerbated by bioaccumulation and formation of highly active metabolites such as MBP, particularly during fetal and early postnatal development.

The current data support that BPA and its analogues are neglected contributors to cancer risk and treatment failure. Reducing the effects of BPA will require coordinated action at multiple levels, including tightening regulations and improving detection and remediation technologies across sources such as soil, water, and food. Overall, one of the most important aspects of dealing with the EDC crisis is recognizing BPA as a global health threat to prevent avoidable cancer morbidity in current and future generations.

## Data Availability

No new data were created or analyzed in this study.

## Abbreviations

The following abbreviations are used in this manuscript:

AKT	Protein Kinase B
AR	Androgen Receptor
ATM	Ataxia-Telangiectasia Mutated
ATP	Adenosine Triphosphate
ATR	ATM and Rad3-Related
BG-1	Human Ovarian Adenocarcinoma Cell Line
BPA	Bisphenol A
BPAF	Bisphenol AF
BPF	Bisphenol F
BPM	Bisphenol M
BPP	Bisphenol P
BPS	Bisphenol S
CAGR	Compound Annual Growth Rate
DNA	Deoxyribonucleic Acid
ECHA	European Chemicals Agency
EGFR	Epidermal Growth Factor Receptor
EMT	Epithelial-to-Mesenchymal Transition
ER	Estrogen Receptor
ERK	Extracellular Signal-Regulated Kinase
ERRγ	Estrogen-Related Receptor Gamma
GPR30	G Protein-Coupled Receptor 30
GPCR	G Protein-Coupled Receptor
GPER	G Protein-Coupled Estrogen Receptor
HIF	Hypoxia-Inducible Factor
HT-29	Human Colorectal Adenocarcinoma Cell Line
ICI	Imperial Chemical Industries
IGF-1R	Insulin-Like Growth Factor 1 Receptor
II	Grade II
III	Grade III
IMR-32	Human Neuroblastoma Cell Line
JCP	Journal of Cancer Prevention
MBP	4-Methyl-2,4-bis(4-hydroxyphenyl)pent-1-ene
MCF-7	Michigan Cancer Foundation-7
MD	Medicine
MDA-MB	MD Anderson–Metastatic Breast Cancer
MMP	Matrix Metalloproteinase
MMP-2	Matrix Metalloproteinase-2
MMP-9	Matrix Metalloproteinase-9
MAPK	Mitogen-Activated Protein Kinase
MYC	c-Myc Oncogene
NSCLC	Non-Small Cell Lung Cancer
NPC	Nasopharyngeal Carcinoma
OVCAR-3	Human Ovarian Cancer Cell Line
PCNA	Proliferating Cell Nuclear Antigen
PKB	Protein Kinase B
PKC	Protein Kinase C
PTEN	Phosphatase and Tensin Homolog
RNA	Ribonucleic Acid
ROS	Reactive Oxygen Species
SH	Sonic Hedgehog
SHH	Sonic Hedgehog
SMA	Smooth Muscle Actin
SK-BR3	HER2-Positive Breast Cancer Cell Line
SK-N	Neuroblastoma Cell Line Family
STAT3	Signal Transducer and Activator of Transcription 3
SUP-B15	Lymphoblastic Leukemia Cell Line
SVHC	Substance of Very High Concern
TDI	Tolerable Daily Intake
TGF	Transforming Growth Factor
TIMP	Tissue Inhibitors of Metalloproteinases
TNBC	Triple-Negative Breast Cancer
USA	United States of America
US	United States
UV	Ultraviolet
VGEF	Vascular Endothelial Growth Factor
YAP	Yes-Associated Protein
ERα	Estrogen Receptor Alpha
ERβ	Estrogen Receptor Beta
PI3K	Phosphoinositide 3-Kinase
mTOR	Mechanistic Target of Rapamycin
HER2	Human Epidermal Growth Factor Receptor 2
NF-κB	Nuclear Factor Kappa B
JAK	Janus Kinase
HIF-1α	Hypoxia-Inducible Factor-1 Alpha
VEGF	Vascular Endothelial Growth Factor
TGF-β	Transforming Growth Factor Beta
FOXO3	Forkhead Box O3
PINK1	PTEN-Induced Kinase 1
SnoN	Ski-Related Novel Protein N
KRAS	Kirsten Rat Sarcoma Viral Oncogene
